# Hiding in plain sight: eating disorders in diverse populations - a case for comprehensive medical education

**DOI:** 10.1186/s40337-024-01174-x

**Published:** 2024-12-30

**Authors:** Tashalee R. Brown, Madeline O. Jansen, Drew Hirsch, Habiba Amir, Alexis E. Duncan, Ginger E. Nicol

**Affiliations:** 1 Department of Psychiatry and Biobehavioral Sciences, University of California Los Angeles, Los Angeles, CA USA; 2 Department of Psychiatry, Washington University School of Medicine, St. Louis, MO USA; 3 Department of Child and Adolescent Psychiatry, Washington University School of Medicine, St. Louis, MO USA; 4 The Brown School, Washington University, St. Louis, MO USA; 5 UCLA National Clinician Scholars Program, 1100 Glendon Ave, Suite 900, Los Angeles, CA 90024 USA

**Keywords:** Racial and ethnic disparities, Lgbtq, Eating disorders, Survey, Medical education

## Abstract

**Background:**

Training gaps regarding the diagnosis and management of eating disorders in diverse populations, including racial, ethnic, sexual, and gender minoritized groups, have not been thoroughly examined.

**Objective:**

This study aimed to examine resident physicians’ knowledge and attitudes regarding eating disorders in diverse populations, with a focus on areas for improved training and intervention.

**Methods:**

Ninety-two resident physicians in internal medicine, emergency medicine, obstetrics/gynecology, psychiatry, and surgery at an academic center completed an online survey from 12/1/2020–3/1/2021, which comprised multiple choice and vignette-style open-ended questions to assess knowledge and attitudes toward the management and clinical presentations of eating disorders. Overall, the survey response rate was 25.7%. Descriptive statistics were reported. Vignette-style questions were analyzed using inductive coding and the frequency of responses was reported.

**Results:**

A minority of resident physicians self-reported confidence in their knowledge of the medical complications (*n* = 42, 45%), risk factors (*n* = 38, 41%), and clinical presentations (*n* = 32, 35%) associated with eating disorders. Responses to vignette-style questions correctly identified relevant management methods (such as electrolyte monitoring and referral to specialty care), but demonstrated limited knowledge of the clinical presentation of eating disorders. Furthermore, most respondents reported a lack of knowledge regarding eating disorders in sexual and gender minoritized patients (*n* = 68, 73.9%) as well as racial and ethnic minoritized patients (*n* = 64, 69.6%).

**Conclusions:**

Our findings suggest concerning gaps in knowledge and confidence among resident physicians with regard to the diagnosis and treatment of eating disorders, particularly in racial, ethnic, sexual, and gender minoritized patients. Moreover, responses to vignette-like questions indicate significant homogeneity in respondents’ perceptions of the clinical presentation of eating disorders, reflecting cultural biases which associate eating disorders with underweight, young, female patients. The majority did not feel competent in treating eating disorders in diverse populations and expressed desire for additional training in this area. More research is needed to better understand and address these gaps in eating disorder training, with the goal of increasing equity in patient outcomes.

## Abstract (plain language summary)

We investigated the knowledge and attitudes of physicians in training regarding eating disorders in diverse populations, including racial, ethnic, sexual, and gender minoritized groups. Our study surveyed 92 physicians in training from various specialties and found concerning gaps in their self-reported understanding and confidence in the diagnosis and treatment of eating disorders, particularly in minoritized populations. The findings of the survey revealed several concerning trends. A significant portion of the resident physicians reported low confidence in their knowledge of the medical complications, risk factors, and clinical presentations of eating disorders. Although they recognized some management methods, they reported limited knowledge about how eating disorders affect racial, ethnic, sexual and gender minoritized patients. Overall, the findings indicate significant gaps in the training of these resident physicians regarding eating disorders, particularly for diverse populations, and many expressed a need for more training to improve their competence in this area. Further research is necessary to better understand and address these gaps to promote equity in outcomes for patients with eating disorders.

## Background

Eating disorders (EDs) are insidious, persistent conditions associated with a significantly elevated risk of medical complications, physical disability, and death [1]. In fact, EDs have the second highest mortality rate of any psychiatric disorder [2]. Although 55.5 million people are affected worldwide, EDs remain under-recognized by clinicians [1]. Racial, ethnic, sexual, and gender minoritized individuals (RESGM) are especially underrepresented in clinical samples [3], yet some studies suggest they are disproportionately affected and have poorer outcomes than other groups [4, 5]. Factors contributing to disparities include lower rates of help seeking and clinician bias [4, 6, 7] Clinician reports and patient accounts suggest a lack of awareness and potential unease among medical professionals regarding diversity-related issues [8, 9]. While interest in ED-related mental health literacy among professionals and patients has increased globally in recent years, especially in North America and Europe, significant gaps remain in the literature, particularly relating to the needs of diverse populations and EDs other than anorexia nervosa and bulimia nervosa [10]. Furthermore, prevailing cultural perceptions of EDs predominantly feature emaciated, rich, white, female individuals, despite the significant heterogeneity in their clinical presentations which is far more reflective of the population at large [5]. These portrayals may dissuade underrepresented populations from seeking care, leading perceptions of EDs among clinicians to predominantly center on the narrow subset of patients who do seek care [1, 5].

Gaps in medical education pertaining to the recognition and subsequent diagnosis of EDs may facilitate the translation of such erroneous perceptions into clinical practice, exacerbating existing medical disparities. Clinical training pertaining to the diagnosis and management of eating disorders is limited in US medical schools [3, 11], and innovative curricula which addresses and/or prevents bias may serve as a key mediator of treatment outcomes in affected minoritized populations. However, the nuances of training gaps regarding EDs in diverse populations have yet to be explored.

## Methods

We investigated physicians’ self-reported knowledge and practices regarding EDs in REGSM populations with an online survey study conducted from 12/1/2020–3/1/2021. Survey questions were generated from a review of existing research and the research team’s previous experience working with eating disorders in diverse populations. The final instrument was composed of questions divided into three domains: knowledge, attitudes, and clinical management practices. Questions were presented in several forms including yes/no, multiple choice, and Likert rating scales. In addition, the survey included several vignettes describing patients with eating disorders, followed by open-ended questions.

The survey was disseminated via email through chief residents or training directors to 334 resident physicians in the following specialties (number respondents/number solicited, response rate): Psychiatry (15/48, 31.25%), Internal Medicine (43/133, 32.33%), Surgery (18/71, 25.35%), Emergency Medicine (9/55, 16.36%), and Obstetrics/Gynecology (7/27, 25.9%). Participants were offered an incentive of being entered into a raffle for three $100 gift cards. Overall, 92 responses were received resulting in an overall response rate of 27.5% (92/334). Data from all specialties were combined as questions did not require specialty level knowledge. Data were analyzed using REDCap software (version 7.1/2019; Vanderbilt University; Nashville TN) [12].

All survey questions were required. Descriptive statistics (frequencies and proportions) were generated for survey responses and reported as a percentage of respondents with a response in each category for a given question. For Likert scale items, “strongly agree” and “agree” were combined to “agree” and “strongly disagree” and “disagree” were combined to “disagree”. Two vignette-based open-ended questions were used to assess physicians’ knowledge of ED management and perceptions of the clinical characteristics of patients with eating disorders. Responses were analyzed using an inductive coding method [13]. Two authors (DH and HA) independently identified codes related to ED management and clinical characteristics of patients with EDs. The leading authors (TB and MJ) reviewed identified codes, consolidated redundancies; final codes were based on discussions until consensus was achieved. The number of physician respondents reporting each code was reported. The study was approved by the Washington University Institutional Review Board.

## Results

### Characteristics of study participants

Participant characteristics are shown in Table 1. Of the 92 respondents, the median age was 29 (range 25–46). The majority of respondents were White (*n* = 59, 58.4%), followed by Asian (*n* = 24, 23.8%). Respondents primarily identified as cisgender female (*n* = 49, 53.3%), followed by cisgender male (*n* = 41, 44.6%). Most respondents identified as heterosexual (*n* = 82, 89.1%). Additionally, few reported receiving training on EDs in their residency program (*n* = 19, 20.7%).

**Table 1:** Characteristics of study participants

Data	Median (range)	*N*	%
**Total respondents**		92	
**Age (years)**	29 (25–46)		
**Race**			
White		59	64.1%
Asian		24	26.1%
Black		*	*
Hispanic		*	*
Middle Eastern or North African		*	*
Native Hawaiian Pacific Islander		*	*
Multiracial		*	*
**Gender**			
Male		41	44.6%
Female		49	53.3%
Non-binary		*	*
**Sexual Identity**			
Heterosexual		82	89.1%
Homosexual		*	*
Bisexual		*	*
Other		*	*
**Training**			
Emergency Medicine		9	16.4%
Internal Medicine		43	32.3%
Obstetrics/Gynecology		7	25.9%
Psychiatry		15	31.3%
Surgery		18	25.4%
**Years in training (years)**	2.2 (1–7)		
Years in training < = 3		70	76.1%
Years in training > 3		22	23.9%
**ED training in residency**:			
Yes		19	20.7%
No		73	79.3%

*Abbreviations* ED = Eating disorder

*Small sample sizes (*n* < 10) are not presented to protect confidentiality

### Knowledge of eating disorder medical management

**Fig. 1:**
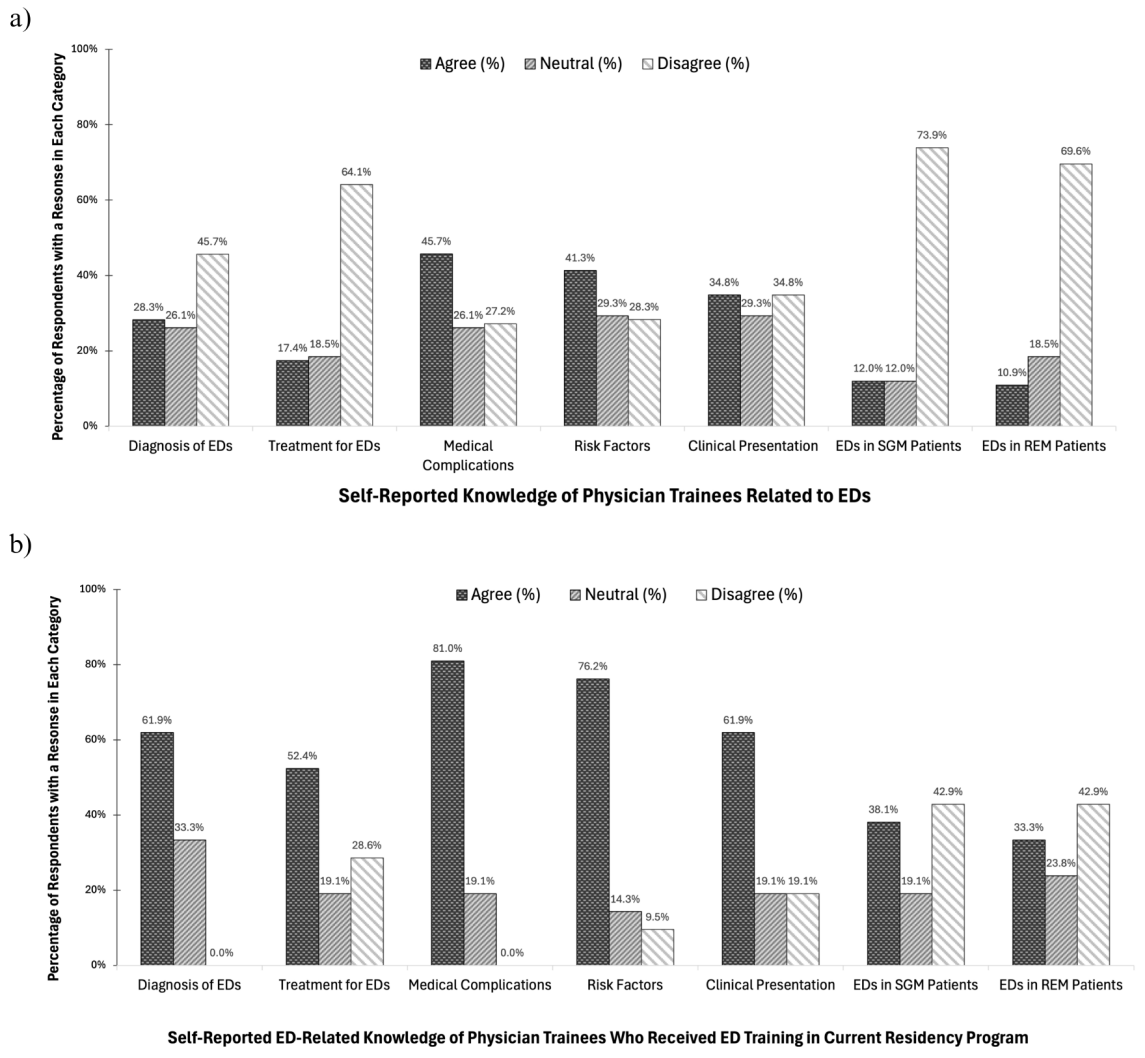
**a** Self-reported knowledge of physician trainees related to EDs (*n* = 92). **b** Self-reported ED-related knowledge of the subset of physician trainees who received ED training in their current residency program (*n* = 21)

Figure 1a illustrates notable findings, including low self-confidence with regards to the diagnosis (*n* = 26, 28%) and treatment (*n* = 16, 17.4%) of EDs. Self-reported confidence in general eating disorder knowledge was highest for medical complications (*n* = 42, 45%), followed by risk factors (*n* = 38, 41%), and their clinical presentations (*n* = 32, 35%). Reported confidence in knowledge of EDs in diverse populations was much lower. Most respondents reported a lack of knowledge with regard to EDs in sexual and gender minoritized patients (*n* = 68, 73.9%) and in racial and ethnic minoritized patients (*n* = 64, 69.6%).

Figure 1b indicates that among respondents who received ED training at their current residency program (*n* = 21, 22.8%), most reported confidence with regards to the diagnosis (*n* = 13, 61.9%) and treatment (*n* = 11, 52.4%) of ED. Confidence was highest with regard to their knowledge of the medical complications (*n* = 17, 81%), risk factors (*n* = 16, 76.2%), and clinical presentations (*n* = 13, 62%) associated with EDs, respectively. Moreover, even among participants who received ED training at their current residency program, only a minority reported confidence in their knowledge of EDs in diverse populations, including sexual and gender minoritized patients (*n* = 8, 38.1%) and racial and ethnic minoritized patients (*n* = 7, 33.3%).

Table 2 indicates a relatively high level of knowledge regarding the medical management of EDs, captured by open-ended responses to vignette-style questions. For the acute management of EDs, respondents most frequently mentioned electrolyte monitoring with a basic or comprehensive metabolic panel (*n* = 81, 88.0%), psychiatric referral/evaluation (*n* = 44, 47.8%), complete blood count (*n* = 34, 37.0%), and screening/monitoring for refeeding syndrome with magnesium (*n* = 21, 22.8%) and phosphorus (*n* = 20, 21.7%). In addition, there is limited diversity in the perceptions of the characteristics of an ED patient (Table 2). Responses were predominantly associated with low body weight/BMI (*n* = 59, 64.1%), psychiatric comorbidities (*n* = 24, 26.1%), female gender (*n* = 19, 20.7%), and young (*n* = 13, 14.1%).

**Table 2:** Vignette-based assessment of knowledge of ED (a) management and (b) characteristics of EDs

**a. Management of EDs codes**	***n***/***N***	%	**Example responses by code** You have identified a patient as having an eating disorder. Describe the workup you would complete (name three).
Electrolytes, metabolic panel, BMP, CMP, Mg, Phos, K	81/92	88.0%	“Electrolytes/BMP”
Psychiatry referral or evaluation	44/92	47.8%	“Psychological assessment including depression and anxiety questionnaires”
CBC	34/92	37.0%	“CBC”
Magnesium (Mg)	21/92	22.8%	“Mg/Phos, CMP”
Phosphorus (Phos)	20/92	21.7%	“CMP, Mg, Phos”
Nutrition referral	18/92	19.6%	“Referral to dietician”
Cardiac/EKG	17/92	18.5%	“EKG”
TSH	10/92	10.9%	“TSH”
**b. Characteristics of ED codes**			What patient characteristics would raise concern for an eating disorder (name three)?
Low weight, low BMI, weight loss, skinny	59/92	64.1%	“emaciated appearance”
Psychiatric comorbidities	24/92	26.1%	“mental health comorbidity”
Female	19/92	20.7%	“female”
Young	13/92	14.1%	“teenage/young adult”
Weight, diet concerns	13/92	14.1%	“significant restriction of food choices or intake”
Binging, Purging	13/92	14.1%	“purging behavior”
High achieving, perfectionistic, competitive	10/92	10.9%	“successful in other areas of their life”
Poor body image	9/92	9.8%	“body image concerns”
Poor dentition	9/92	9.8%	“tooth decay in a young patient”
High weight, weight gain, obese	4/92	4.3%	“morbid obesity”

*Abbreviations* BMI = body mass index, BMP = Basic metabolic panel, CBC = complete blood count, CMP = comprehensive metabolic panel, ED = Eating disorder, EKG- electrocardiogram, Mg = Magnesium, Phos = Phosphorus TSH = thyroid stimulating hormone

### Attitudes and practices towards patients with eating disorders

**Fig. 2:**
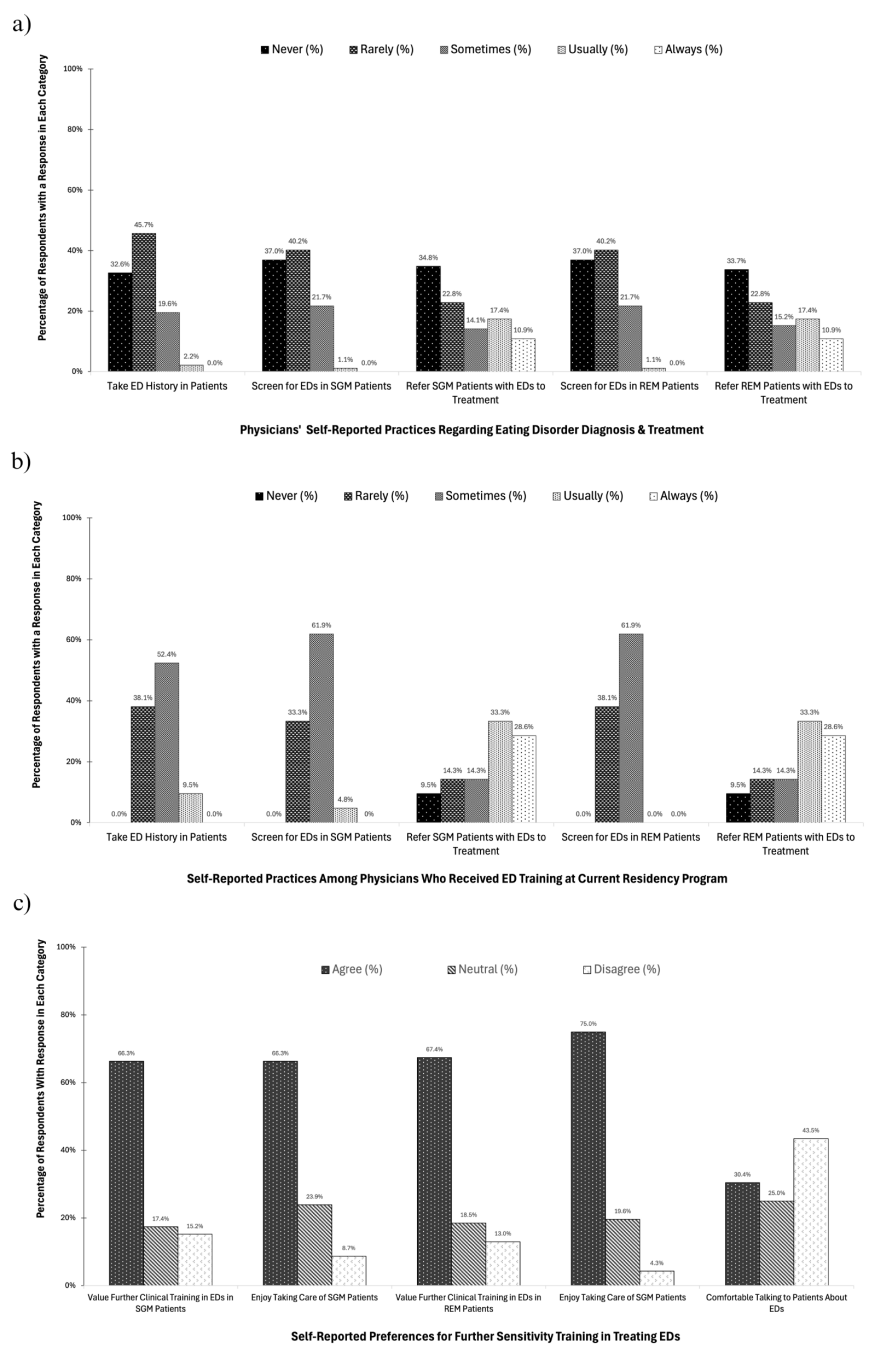
**a** Physicians’ self-reported practices regarding diagnosis and treatment of EDs in general and in RESGM populations (*n* = 92). **b** Self-reported practices related to ED diagnosis and management among physicians who received ED training at their current residency program (*n* = 21). **c** Self-reported preferences for further sensitivity training regarding the treatment of EDs in diverse populations (*n* = 92)

Figure 2a denotes that few respondents feel comfortable talking with patients about ED behaviors (*n* = 28, 30%). In addition, many indicated that they never (*n* = 34, 33%) or rarely (*n* = 37, 40%) take an ED history in their patients. Moreover, among racial and ethnic minoritized patients, several respondents reported they either never (*n* = 34, 37%) or rarely (*n* = 38, 41%) screen for EDs. Similarly, many indicated they never (*n* = 31, 34%) or rarely (*n* = 21, 23%) refer these patients to treatment. Comparable patterns were observed with sexual and gender minoritized patients, with many stating they never (*n* = 34, 37%) or rarely (*n* = 37, 40%) conduct screenings and most noting they never (*n* = 32, 35%) or rarely (*n* = 21, 23%) refer these patients with EDs to treatment.

Figure 2b indicates that most participants who received ED training at their current residency programs (*n* = 21, 22.8%) reported feeling comfortable talking to their patients about ED-related behaviors (*n* = 16, 76.2%). Additionally, few respondents who received ED training reported that they rarely (*n* = 8, 38%) or never (*n* = 0, 0%) take an ED history in their patients. In sexual and gender minoritized patients, few participants of this group indicated that they rarely (*n* = 7, 33.3%) or never (*n* = 0, 0%) screen for EDs. Likewise, few of these respondents indicated that they rarely (*n* = 3, 14.3%) or never (*n* = 2, 9.5%) refer these patients with EDs to treatment. In addition, among racial and ethnic minoritized patients, few respondents of this group indicated that they rarely (*n* = 8, 38.1%) or never (*n* = 0, 0%) screen for EDs. Similarly, a minority of respondents indicated that they rarely (*n* = 3, 14.3%) or never (*n* = 2, 9.5%) refer racial and ethnic minoritized patients with eating disorders to treatment.

Figure 2c shows that most respondents (*n* = 61, 66.3%) reported that they enjoy working with patients from sexual and gender minoritized backgrounds and would value further clinical training pertaining to the diagnosis and management of EDs in this population. Similarly, most respondents (*n* = 69, 75.0%) reported that they enjoyed working with patients from racial and ethnic minoritized backgrounds, and would value further clinical training with regards to EDs in this population. Lastly, few respondents reported feeling comfortable talking with patients about ED behaviors (*n* = 28, 30%).

### Perceived barriers to access to mental healthcare

**Fig. 3:**
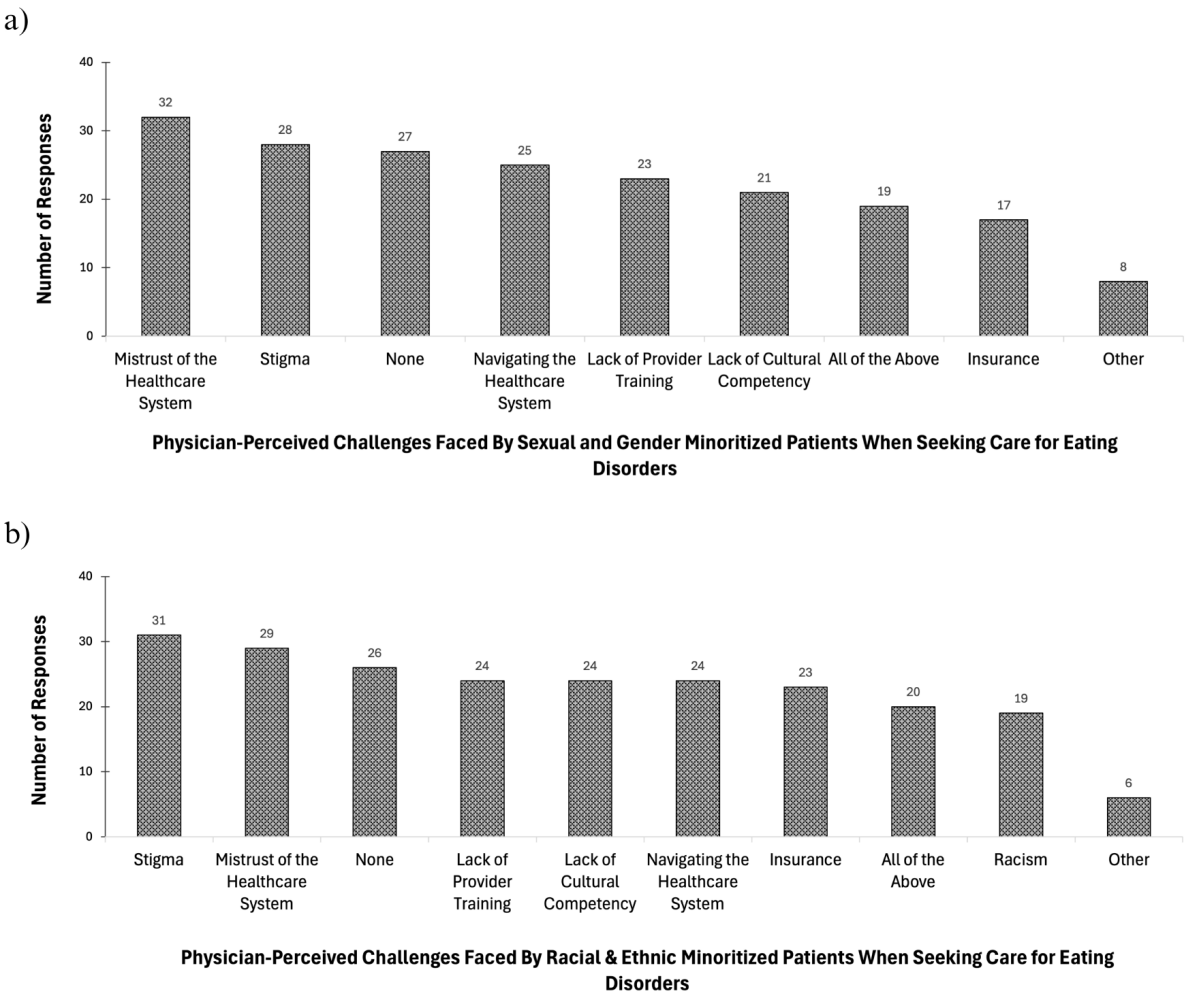
Resident-reported perceived barriers faced by **a** racial and ethnic minoritized, **b** sexual and gender minoritized individuals receiving care for EDs

Figure 3a demonstrates that the top two barriers for sexual and gender minoritized patients seeking ED treatment perceived by resident trainees were “mistrust of the healthcare system,” selected (*n* = 32, 35%), followed by “stigma”, selected (*n* = 28, 30%) times. With regard to racial and ethnic minoritized patients, Fig. 3b illustrates that the top two barriers for racial and ethnic minoritized patients seeking ED treatment perceived by resident trainees were “stigma”, selected (*n* = 31, 34%) and “mistrust of the healthcare system”, selected (*n* = 29, 32%) times. The third highest barrier selected by trainees was “none”, selected (*n* = 27, 30%) times.

## Discussion

Recent US Preventive Services Task Force recommendations call for additional research to reduce ED disparities by improving detection among REGSM patients [14]. This study attempts to evaluate ED populations from the perspective of medical education. This study is unique among others on the topic because it asks questions directly to trainees and involves a diversity of medical subspecialties. The survey collected qualitative data that provides in-depth responses with context, particularly concerning the gap between medical knowledge and experience. Most importantly, this study provides useful information that can guide curriculum development to reduce bias. As such, this study provides strong contributions to this area of need in clinical research.

Our findings suggest a concerning disparity in the knowledge and confidence levels of resident physicians regarding EDs, particularly in diverse populations. While respondents showed some confidence in general knowledge about EDs, particularly with regard to the medical management of EDs, there was a notable lack of confidence with regard to the diagnosis and treatment of EDs in REGSM patients. These findings resonate with recent research suggesting inadequate knowledge and stigma surrounding EDs among physicians and trainees [15, 16]. Such a gap in training and awareness may contribute to delays in diagnosis and treatment, potentially exacerbating the challenges faced by patients with EDs, particularly in diverse populations who may be less likely to seek out care [1, 5].

It is well documented that racial and ethnic minoritized individuals, as well as men, are less likely to seek help for EDs [4, 17, 18]. For example, per the 2012–2013 National Epidemiologic Survey on Alcohol and Related Conditions-III (*N* = 36,309), Black and Hispanic/Latino individuals are statistically significantly less likely to seek help for EDs compared to White individuals [17]. Additionally, despite similar prevalence rates based on pooled data from the NIMH Collaborative Psychiatric Epidemiological Surveys (CPES), ethnic minority groups are less likely to utilize mental health services for EDs [18].

It is also known that sexual and gender minoritized individuals exhibit higher rates of EDs compared to their heterosexual and cisgender counterparts [19]. Transgender individuals, in particular, have significantly higher odds of self-reported EDs and compensatory behaviors compared to cisgender heterosexual peers [19]. Transgender patients often report negative experiences with healthcare providers, including unwelcome comments about their bodies and gender, which can deter them from seeking care [20].

Moreover, vignette-like questions revealed significant homogeneity in resident physicians’ perceptions of the clinical presentation of EDs, which aligns with cultural perceptions of EDs that ultimately enable EDs in diverse populations to go unnoticed [1, 5]. Despite a majority of participants mentioning weight loss or malnourishment as a feature of the clinical presentation of EDs, very few (*n* = 4, 4.3%) mentioned weight gain or a high weight. Such predilections exclude a large proportion of patients with EDs, such as those with restrictive eating disorders at normal or high weights, as well as bulimia nervosa, binge eating disorder, avoidant/restrictive food intake disorder (ARFID), and other specified feeding and eating disorders (OSFED) [8, 21]. When such characteristics were mentioned, terms such as “morbid obesity” were used, implying that a more severe physiological manifestation of illness should be present for an ED diagnosis to be considered. While weight has clinical utility in the diagnosis of EDs, there is growing concern that it is relied on too heavily as an all-inclusive measure of health, leading to missed or delayed diagnoses [22].

Likewise, respondents mentioned other characteristics representative of the overlying culturally-sustained cognitive prototype of EDs. Participants exhibited a gender bias toward young, female patients. Presentations of EDs in men are historically characterized as “rare” and “atypical”, which can delay recognition and help-seeking behaviors in this population [23]. Clinicians may also be less likely to consider or recognize EDs in men, as men are historically underrepresented in eating disorder research and thus, healthcare providers may be unaware of more male-specific ED presentations [23]. For instance, men with EDs may present with a focus on muscularity rather than thinness, a distinction that many existing diagnostic tools inadequately address [24].

Respondents reported that the top two physician-perceived barriers experienced by REGSM were mistrust of the healthcare system and stigma. Interestingly, the third highest response reported was a lack of barrier. Several respondents additionally reported that they would value more training in REGSM populations and that they enjoy taking care of these populations.

Notably, the subset of participants who completed ED-specific training in their current residency program, while relatively small in number, showed greater confidence on all measures pertaining to the diagnosis, treatment, and general knowledge regarding EDs. In addition, they were more likely to report greater frequencies of proactive practices, such as taking ED histories in their patients, screening for EDs in REGSM patients, and referring REGSM patients with EDs to treatment. Structured ED-specific training in residencies may therefore show efficacy in increasing recognition of EDs and reducing ED-related burdens on diverse populations, particularly when it emphasizes diversity in how EDs may present. By fostering a greater understanding of the unique challenges faced by REGSM individuals with EDs, training programs may help reduce the impact of barriers (i.e. stigma) and improve referral pathways, ultimately enhancing the likelihood of timely and appropriate interventions.

In order to contextualize study findings, it is important to state that several physician organizations have developed recommendations and practice guidelines for managing EDs. Many of these specialties are included in this study, including the American Psychiatric Association [21], the American Association of Child and Adolescent Psychiatry [25], the American College of Obstetricians and Gynecologists [26], and the American Medical Society for Sports Medicine [27]. While Emergency Medicine did not have practice guidelines, there is an article by Trent et al. [28] which reviewed ED management in the Emergency Department. Of note, the majority of these guidelines have focused on acute medical presentation, management, and risk factors for EDs, and do not provide guidance on treating populations marginalized by race, ethnicity, sexuality, and gender.

This study is subject to limitations, including limited sample size at a single institution, limiting stratified analysis by specialty. The relatively low response rate also presents a limitation that may restrict the generalizability of its findings due to possible response bias of our sample. Moreover, significance testing was not conducted, as the study aimed to describe trends regarding perception and attitudes among physicians in training rather than establish statistical relationships limited by a small sample size. Further study of resident physicians’ knowledge, skills, attitudes, and behaviors is needed, and will be critically important in devising interventions aimed to specifically address the needs of RESGM patients with EDs.

Taken together, this study’s results and the lack of emphasis on diverse populations in ED physician practice guidelines highlight a lack of knowledge of treating EDs in RESGM patients and may indicate a need for more focused research in this area. Our qualitative results suggest the potential presence of biases among trainees, which may serve to promote disparities in care among RESGM patients. This finding provides more evidence for the potential benefit of improved medical training. While several trainees report understanding of the impact that stigma and mistrust of the healthcare system can have among RESGM patients, several also report a lack of awareness of any barriers experienced by these patients. This lack of awareness may drive disparities in care among RESGM patients and may successfully be addressed with improved ED training [9, 29, 30]. Lastly, study results suggest the acceptability for more training among RESGM for EDs.

## Data Availability

No datasets were generated or analysed during the current study.

## References

[1] Santomauro DF, Melen S, Mitchison D, Vos T, Whiteford H, Ferrari AJ. The hidden burden of eating disorders: an extension of estimates from the global burden of Disease Study 2019. Lancet Psychiatry. 2021;8(4):320–8. 10.1016/S2215-0366(21)00040-7. 33675688 10.1016/S2215-0366(21)00040-7 PMC7973414

[2] Chesney E, Goodwin GM, Fazel S. Risks of all-cause and suicide mortality in mental disorders: a meta-review. World Psychiatry. 2014;13(2):153–60. 10.1002/wps.20128. 24890068 10.1002/wps.20128 PMC4102288

[3] Solmi F, Bould H, Lloyd EC, Lewis G. The shrouded visibility of eating disorders research. Lancet Psychiatry. 2021;8(2):91–2. 10.1016/S2215-0366(20)30423-5. 33227239 10.1016/S2215-0366(20)30423-5 PMC7613242

[4] Sim L. Our eating disorders Blind Spot: sex and Ethnic/Racial disparities in help-seeking for eating disorders. Mayo Clin Proc. 2019;94(8):1398–400. 10.1016/j.mayocp.2019.06.006. 31378224 10.1016/j.mayocp.2019.06.006

[5] Halbeisen G, Brandt G, Paslakis G. A Plea for diversity in eating disorders Research. Front Psychiatry. 2022;13:820043. 10.3389/fpsyt.2022.820043. Published 2022 Feb 18. 35250670 10.3389/fpsyt.2022.820043 PMC8894317

[6] Jansen MO, Brown TR, Xu KY, Glowinski AL. Using Digital Technology to overcome racial disparities in Child and Adolescent Psychiatry. J Am Acad Child Adolesc Psychiatry. 2022;61(10):1211–7. 10.1016/j.jaac.2022.03.013. 35358663 10.1016/j.jaac.2022.03.013 PMC9970009

[7] Brown TR, Xu KY, Glowinski AL. Cognitive behavioral therapy and the implementation of antiracism. JAMA Psychiatry. 2021;78(8):819–20. 10.1001/jamapsychiatry.2021.0487. 33950160 10.1001/jamapsychiatry.2021.0487 PMC9924160

[8] Sonneville KR, Lipson SK. Disparities in eating disorder diagnosis and treatment according to weight status, race/ethnicity, socioeconomic background, and sex among college students. Int J Eat Disord. 2018;51(6):518–26. 10.1002/eat.22846. 29500865 10.1002/eat.22846

[9] Becker AE, Hadley Arrindell A, Perloe A, Fay K, Striegel-Moore RH. A qualitative study of perceived social barriers to care for eating disorders: perspectives from ethnically diverse health care consumers. Int J Eat Disord. 2010;43(7):633–47. 10.1002/eat.20755. 19806607 10.1002/eat.20755 PMC3020364

[10] Bullivant B, Rhydderch S, Griffiths S, Mitchison D, Mond JM. Eating disorders mental health literacy: a scoping review. J Mental Health. 2020;29(3):336–49. 10.1080/09638237.2020.1713996 32041463

[11] Mahr F, Farahmand P, Bixler EO, et al. A national survey of eating disorder training: National Survey of eating disorder training. Int J Eat Disord. 2015;48(4):443–5. 10.1002/eat.22335. 25047025 10.1002/eat.22335

[12] Harris PA, Taylor R, Thielke R, Payne J, Gonzalez N, Conde JG. Research electronic data capture (REDCap) – a metadata-driven methodology and workflow process for providing translational research informatics support. J Biomed Inf. 2009;42(2):377–81. 10.1016/j.jbi.2008.08.010 PMC2700030 18929686

[13] Braun V, Clarke V. Using thematic analysis in psychology. Qualitative Res Psychol. 2006;3(2):77–101. 10.1191/1478088706qp063oa.

[14] US Preventive Services Task Force, Davidson KW, Barry MJ, et al. Screening for eating disorders in adolescents and adults: US Preventive Services Task Force Recommendation Statement. JAMA. 2022;327(11):1061–7. 10.1001/jama.2022.1806. 35289876 10.1001/jama.2022.1806

[15] Currin L, Waller G, Schmidt U. Primary care physicians’ knowledge of and attitudes toward the eating disorders: do they affect clinical actions? Int J Eat Disord. 2009;42(5):453–8. 10.1002/eat.20636. 19115367 10.1002/eat.20636

[16] Ma C, Gonzales-Pacheco D, Cerami J, Coakley KE. Emergency medicine physicians’ knowledge and perceptions of training, education, and resources in eating disorders. J Eat Disord. 2021;9(1):4. 10.1186/s40337-020-00355-8. Published 2021 Jan 6. 33407918 10.1186/s40337-020-00355-8 PMC7789763

[17] Coffino JA, Udo T, Grilo CM. Rates of Help-Seeking in US Adults With Lifetime DSM-5 Eating Disorders: Prevalence Across Diagnoses and Differences by Sex and Ethnicity/Race. Mayo Clin Proc. 2019;94(8):1415–1426. 10.1016/j.mayocp.2019.02.030 10.1016/j.mayocp.2019.02.030 PMC6706865 31324401

[18] Comparative, Prevalence, Marques L, Alegria M, Becker AE, et al. Int J Eat Disord. 2011;44(5):412–20. 10.1002/eat.20787. 20665700 10.1002/eat.20787 PMC3011052

[19] Ferrero EM, Yunker AG, Cuffe S, et al. Nutrition and Health in the Lesbian, Gay, Bisexual, Transgender, Queer/Questioning Community: a narrative review. Adv Nutr. 2023;14(6):1297–306. 10.1016/j.advnut.2023.07.009. 37536566 10.1016/j.advnut.2023.07.009 PMC10721458

[20] Hartman-Munick SM, Silverstein S, Guss CE, Lopez E, Calzo JP, Gordon AR. Eating disorder screening and treatment experiences in transgender and gender diverse young adults. Eat Behav. 2021;41:101517. 10.1016/j.eatbeh.2021.101517. 33962139 10.1016/j.eatbeh.2021.101517 PMC9645530

[21] American Psychiatric Association. Diagnostic and Statistical Manual of Mental Disorders. 5th ed. Washington D.C.: 2013.

[22] Ramaswamy N, Ramaswamy N. Overreliance on BMI and Delayed Care for Patients With Higher BMI and Disordered Eating. AMA J Ethics. 2023;25(7):E540-E544. Published 2023 Jul 1. 10.1001/amajethics.2023.540 10.1001/amajethics.2023.540 37432007

[23] Murray SB, Nagata JM, Griffiths S, et al. The enigma of male eating disorders: a critical review and synthesis. Clin Psychol Rev. 2017;57:1–11. 10.1016/j.cpr.2017.08.001. 28800416 10.1016/j.cpr.2017.08.001

[24] Halbeisen G, Laskowski N, Brandt G, Waschescio U, Paslakis G. Eating disorders in men. Dtsch Arztebl Int. 2024;121(3):86–91. 10.3238/arztebl.m2023.0246. 38019152 10.3238/arztebl.m2023.0246 PMC11002438

[25] Lock J, La Via MC, American Academy of Child and Adolescent Psychiatry (AACAP) Committee on Quality Issues (CQI). Practice parameter for the assessment and treatment of children and adolescents with eating disorders. J Am Acad Child Adolesc Psychiatry. 2015;54(5):412–25. 10.1016/j.jaac.2015.01.018. 25901778 10.1016/j.jaac.2015.01.018

[26] ACOG. Gynecologic Care for Adolescents and Young Women With Eating Disorders. Accessed June 16. 2023. https://www.acog.org/en/clinical/clinical-guidance/committee-opinion/articles/2018/06/gynecologic-care-for-adolescents-and-young-women-with-eating-disorders

[27] Chang CJ, Putukian M, Aerni G, et al. Mental Health issues and psychological factors in athletes: detection, management, effect on performance, and Prevention: American Medical Society for sports Medicine position Statement. Clin J Sport Med. 2020;30(2):e61–87. 10.1097/JSM.0000000000000817. 32000169 10.1097/JSM.0000000000000817

[28] Trent SA, Moreira ME, Colwell CB, Mehler PS, editors. ED management of patients with eating disorders. *Am J Emerg Med*. 2013;31(5):859–865. 10.1016/j.ajem.2013.02.035 10.1016/j.ajem.2013.02.035 23623238

[29] Mikulak M. For whom is ignorance bliss? Ignorance, its functions and transformative potential in trans health. J Gend Stud. 2021;30(7):819–29. 10.1080/09589236.2021.1880884.

[30] Becker AE, Franko DL, Speck A, Herzog DB. Ethnicity and differential access to care for eating disorder symptoms. Int J Eat Disord. 2003;33(2):205–12. 10.1002/eat.10129. 12616587 10.1002/eat.10129

